# Human Serum Albumin as a Prodrug Carrier for Tumor Therapy: Mechanisms, Applications, and Future Perspectives

**DOI:** 10.3390/pharmaceutics18050557

**Published:** 2026-04-30

**Authors:** Yuhong Shang, Shuangran Wang, Yingyi Yan, Encheng Tian, Lan She, Zhiqiang Ma

**Affiliations:** 1 School of Pharmacy, Naval Medical University, Shanghai 200433, China; 2 School of Nursing, Shanghai Jiguang Polytechnic College, Shanghai 201901, China

**Keywords:** human serum albumin (HSA), prodrug delivery, tumor-targeted therapy, nanocarriers, combination therapy

## Abstract

Human serum albumin (HSA), as a natural protein carrier, possesses excellent biocompatibility and drug binding capacity. Due to the synergistic effects of the enhanced permeability and retention (EPR) effect and Gp60/SPARC-mediated active targeting, this drug carrier demonstrates favorable tumor selectivity and can be enriched in tumor tissues to achieve long-term therapeutic effects. Particularly, HSA undergoes pH-dependent recycling through the neonatal Fc receptor (FcRn), which significantly prolongs its half-life and enhances its feasibility as a drug delivery platform. In practical clinical applications, the regulation of HSA release rates requires multiple strategies to work synergistically. Additionally, the targeting efficiency of delivery systems due to tumor heterogeneity remains a major bottleneck limiting its universality. This article systematically reviews the unique advantages, clinical applications, challenges, and future perspectives of HSA as a prodrug carrier in tumor therapy.

## 1. Introduction

According to data released by the World Health Organization, approximately 19.3 million new malignant tumor cases and 10 million new deaths occurred globally in 2020, making cancer one of the leading causes of death worldwide [1]. It is estimated that by 2050, there will be over 35 million new cancer cases [2]. With the gradual aggravation of population aging, the burden of major chronic diseases, including malignant tumors, is on the rise [3]. The incidence rates of different cancer types show spatial distribution differences, and the prevention and control situation is severe. Refractory solid tumors refer to malignant solid tumors that are difficult to control by conventional means, commonly seen in pancreatic cancer, melanoma, liver cancer, and advanced colon cancer. Clinically, due to their complex pathogenesis, high invasiveness and metastasis, resistance to treatment, or multidrug resistance, they have become one of the main challenges in the field of tumor therapy. Although various treatment modalities for malignant tumors have developed significantly in recent years [4,5,6], the chemotherapy methods currently used in clinical practice are effective in controlling primary tumor sites. Notably, evidence shows that patients still develop distant metastases even when local disease is effectively controlled, indicating that current mainstream treatment modalities remain insufficient [7,8]. Traditional tumor therapeutic agents (such as chemotherapeutic drugs) have many limitations in clinical applications. Due to their nonspecific actions in vivo, direct use can cause damage to surrounding normal tissue cells while destroying cancer cells [9]. Although chemically targeted drugs (such as vorinostat and dasatinib) have certain tumor-targeting potential, they are likely to be degraded during the process of reaching target sites, causing damage to surrounding tissues with significant toxic side effects. These drugs are often accompanied by high adverse event rates (patients frequently develop diarrhea, rashes, itching, acne, and other symptoms), limited treatment efficacy in advanced patients, and the development of tumor drug resistance.

Drug delivery carriers leverage their binding advantages to alleviate adverse reactions during the drug targeting process by combining effective drug components with themselves and delivering them to specific locations at appropriate concentrations and pH levels [10]. In recent years, with the interdisciplinary development of nanotechnology and biomaterials science, targeted therapeutic strategies based on nanocarrier delivery systems have provided new insights for precise tumor treatment. The prodrug strategy is an effective approach used to significantly improve drug pharmacokinetic properties and reduce toxic side effects. By chemically modifying active drugs into inactive or low-activity precursor forms, these prodrugs release the active parent drug under specific conditions in vivo to achieve therapeutic effects. However, the prerequisite for achieving efficient prodrug delivery is selecting an appropriate carrier: an ideal carrier must possess biocompatibility, prolonged circulation half-life, tumor-targeting ability, and high drug-loading capacity. In this context, human serum albumin (HSA), as a natural biocompatible carrier, is an extremely promising carrier choice due to its unique biological characteristics and functions, avoiding potential immune responses and toxicity associated with non-natural materials.

In recent years, research on HSA-based nanocarriers in tumor therapy has made significant progress. Through nanotechnology, HSA can be designed as a drug delivery system with targeting functions, improving the selectivity of anti-tumor drugs and reducing their toxic side effects. This review discusses the structure and advantages of human serum albumin, binding strategies, and clinical applications and finally provides an outlook on the challenges and future directions of HSA in the field of tumor therapy, aiming to establish a theoretical foundation for new tumor treatment approaches using HSA as a prodrug carrier.

## 2. Biological Characteristics and Advantages of Human Serum Albumin

**Figure 1:**
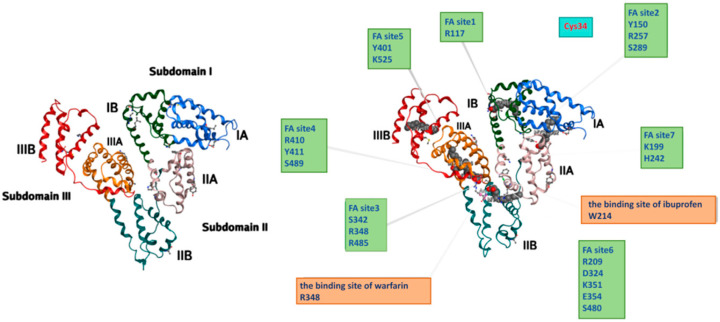
Schematic diagram of human serum albumin structure and related binding sites. Human serum albumin (HSA) is composed of three homologous domains (I, II, III), each further divided into two subdomains (A and B). This heart-shaped conformation contains multiple functional binding pockets, including the major drug-binding sites Sudlow Site I (warfarin-binding site) and Sudlow Site II (ibuprofen-binding site), seven fatty acid (FA) binding sites, and the free cysteine residue Cys34. The diagram illustrates the spatial distribution of these critical sites and key amino acid residues, highlighting HSA’s multi-ligand binding capacity for drugs, fatty acids, and other small molecules.

Human serum albumin is the most abundant protein in plasma, synthesized as a precursor compound by hepatocytes in the liver [11]. After maturation, this precursor compound is secreted into the vascular lumen [12]. Mature human serum albumin consists of 585 amino acids and possesses a series of repeated six-helix subdomains [13,14]. The α-helix secondary structure constitutes the highest proportion, approximately 67%, which, through coiling and folding, forms a specific heart-shaped conformation [15,16]. High-resolution X-ray crystal structures reveal three major regions in albumin: Domain I, Domain II, and Domain III (Figure 1 and Table 1). Each domain is further subdivided into two subregions, A and B, which form a cylindrical structure through relatively oriented grooves. This structural design not only endows HSA with certain flexibility but also enables it to bind to various small molecular substances and transport them to tissues throughout the body, such as fatty acids, metal ions, and drugs [17].

In the amino acid sequence of HSA, there are 35 cysteine residues. Among them, 34 cysteine residues form 17 pairs of intramolecular disulfide bonds through oxidation reactions. These disulfide bonds act like “molecular rivets,” providing stability and rigidity to the three-dimensional structure of HSA, ensuring it maintains the correct folding state in physiological environments [18,19]. Additionally, HSA has a unique free cysteine residue (Cys-34) with a free thiol group (-SH), which remains in a reduced state under physiological conditions and exhibits antioxidant properties. This thiol group is a key site for HSA’s participation in redox reactions [20]. The human albumin structure also contains hydrophobic pocket structures with positively charged lysine and arginine residues, located in the A subregions of subdomain II and subdomain III, namely subdomain IIa and subdomain IIIa. These are important binding sites for hydrophobic anticancer drugs, also named Sudlow Site I and Sudlow Site II [21]. Specifically, Site I favors larger heterocyclic anions, represented by the drug warfarin; hence, Site I is also called the warfarin site. Site II is a site with ibuprofen as a typical ligand, also known as the benzodiazepine site [22]. In addition to the aforementioned hydrophobic drug binding sites, HSA also has multiple active binding sites, such as fatty acid binding sites. Under normal physiological pH conditions, the surface of human serum albumin contains numerous anionic groups, exhibiting high negative charge density, which enhances its solubility and drug interaction capabilities [23]. Therefore, HSA demonstrates multi-affinity for endogenous and exogenous small molecular compounds, as well as a series of peptide fragments and proteins [24]. For example, the fatty acid binding site (FA1) can specifically recognize and bind to heme, participating in heme clearance in the body [25].

**Figure 2:**
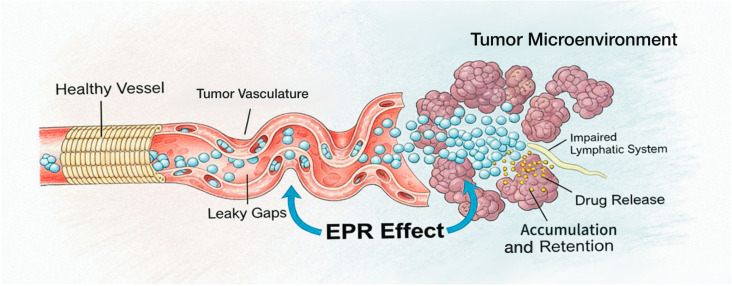
EPR effect in tumor tissues. HSA–based prodrug nanoparticles extravasate from the hyperpermeable tumor vasculature into the tumor interstitium and accumulate in the tumor microenvironment due to impaired lymphatic drainage, thereby achieving passive targeting and long-term retention.

As a natural drug delivery carrier, HSA possesses a combination of high biocompatibility and good biodegradability [26], making its advantages particularly prominent in tumor therapy. The vascular structure of tumor tissues differs from that of normal tissues, with larger gaps between vascular endothelial cells and high permeability, facilitating the penetration of drugs or nanoparticles from blood vessels into tumor tissues. Meanwhile, the lymphatic system in tumor tissues is underdeveloped, lacking effective lymphatic drainage, which further promotes drug accumulation in tumor tissues. Prodrug nanoparticles based on human serum albumin (HSA) generally have a hydrodynamic diameter between 10 and 200 nm. They can passively extravasate into the tumor interstitium through the hyperpermeability of tumor vessels and impaired lymphatic drainage, thereby achieving long-term retention in the tumor microenvironment and realizing targeted enrichment in tumor tissues. This is known as the enhanced permeability and retention (EPR) effect of tumors. [27] (Figure 2).

In addition to passive targeting, HSA can also achieve active targeting by binding to specific receptors on the surface of tumor cells. Research indicates that drugs using HSA as a carrier can be transported across endothelial cells to target tissues through HSA-binding proteins. Multiple receptors capable of binding to HSA exist on the surface of tumor cells, such as Gp60. Among them, Gp60, also known as albondin, is a glycoprotein highly expressed on the surface of tumor vascular endothelial cells. It can specifically recognize and bind to HSA, mediating HSA entry into cells through endocytosis. Subsequently, HSA and its carried drugs can be transported from vascular endothelial cells into tumor tissues through intercellular transport [28].

**Figure 3:**
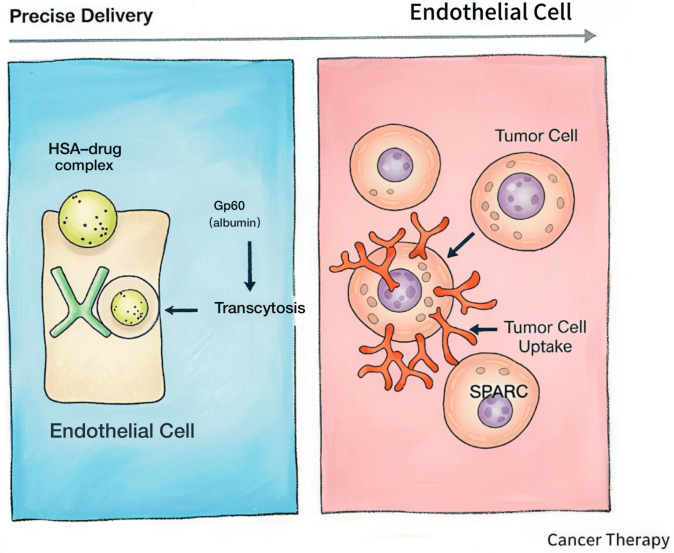
Active targeting mechanism mediated by Gp60 and SPARC.HSA-drug complexes cross the endothelial barrier via Gp60-mediated transcytosis. After extravasation into the tumor microenvironment, the complexes are taken up by tumor cells with the assistance of SPARC (secreted protein acidic and rich in cysteine), which enhances the targeted accumulation and intracellular delivery of albumin-based therapeutics at tumor sites.

In addition to trans-endothelial transport mediated by Gp60, SPARC also plays a vital role in the active targeting of albumin. SPARC (secreted protein acidic and rich in cysteine), also known as osteonectin, is an important matricellular factor that plays a key regulatory role in tissue remodeling and homeostasis. Notably, SPARC exhibits dual biological functions in different tumor types. The extracellular matrix can directly mediate cellular signal crosstalk through receptors such as integrins, while SPARC deficiency results in abnormal extracellular matrix structure and further disturbs downstream signal transduction. In mouse models, SPARC knockout significantly accelerates tumor growth and enhances metastatic potential, suggesting that SPARC can affect tumor progression and metastasis by regulating the deposition and composition of the extracellular matrix. Therefore, in specific tumor types, SPARC can participate in the tumor-targeted accumulation and intracellular delivery of albumin (HSA)-based nanoparticles through its matrix-regulating and signal-mediating functions [29]. Beyond the transmembrane transport mediated by Gp60, another critical component in the active targeting machinery of HSA is the matricellular protein SPARC. Through active targeting mechanisms, HSA-based prodrugs can more precisely act on tumor cells, improving drug targeting and efficacy. Research indicates that Gp60 and SPARC cooperate to facilitate Abraxane (albumin-bound paclitaxel) entry into tumor sites [30] (Figure 3).

**Figure 4:**
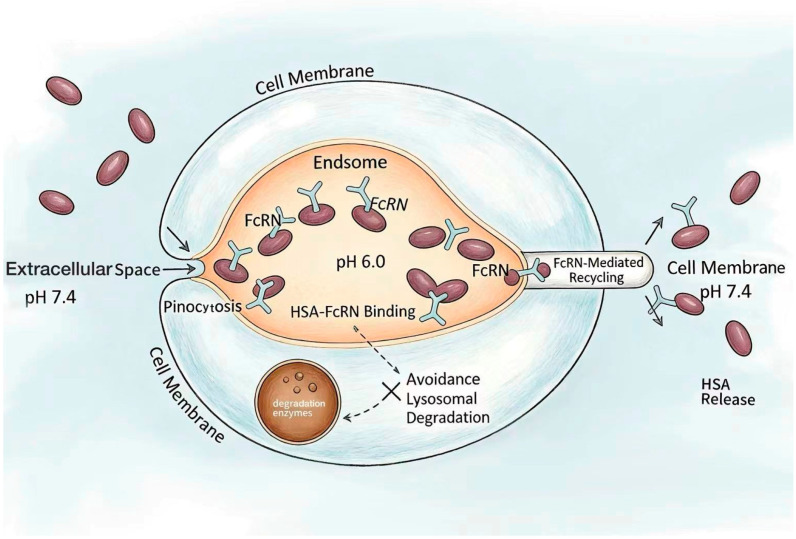
FcRn-mediated recycling mechanism. HSA enters cells via pinocytosis and binds to the neonatal Fc receptor (FcRn) in acidic endosomes (pH 6.0). The HSA–FcRn complex is then recycled to the cell membrane, where the neutral extracellular pH (7.4) triggers dissociation and release of HSA. This mechanism protects HSA from lysosomal degradation and significantly prolongs its half-life in vivo.

Notably, the active targeting property of HSA is synergistically linked to its prolonged half-life: while active targeting promotes the specific accumulation and cellular uptake of HSA–drug complexes in tumor tissues, the FcRn-mediated recycling mechanism significantly extends their retention time in vivo, allowing the drug sufficient time to exert therapeutic effects at the target site. In the body, the average half-life of human serum albumin is approximately three weeks, which greatly improves pharmacokinetics and promotes interactions between drugs and the body [31]. This long half-life mechanism depends on the interaction between HSA and cellular receptors (namely the neonatal Fc receptor, FcRn) and the recycling of internalized albumin [32,33]. Studies show that the neonatal Fc receptor (FcRn) recycled by cells transfers albumin from lysosomal degradation through endosomal recycling pathways [33], thus protecting albumin from lysosomal degradation. HSA enters cells via pinocytosis and binds FcRn in acidic endosomes; the complex is then recycled to the cell membrane and released at physiological pH [34,35,36] (Figure 4). Therefore, this binding and release mechanism is based on strict pH-dependent binding between FcRn and HSA. Through the FcRn-mediated recycling mechanism, the half-life of HSA is significantly prolonged, increasing its applicability as a drug carrier.

## 3. Applications of HSA-Based Prodrugs in Tumor Therapy

### 3.1. Chemotherapy Prodrugs Based on HSA

Chemotherapy is a treatment method that uses chemical drugs to kill or inhibit the growth of cancer cells in the body, widely applied in the comprehensive treatment of various malignant tumors. HSA-based chemotherapy prodrugs involve conjugating chemotherapeutic agents to HSA through chemical bonds or other methods, forming a precursor drug that can stably exist in vivo and can release the chemotherapeutic agent under specific conditions (such as reaching tumor tissues) to exert therapeutic effects [37]. They are characterized by enhanced tumor targeting, improved pharmacokinetic properties of the drug, and reduced toxic side effects [37,38,39].

As a natural endogenous carrier, albumin provides a universal strategy for the optimized delivery of various chemotherapeutic agents, among which the development of paclitaxel formulations is the most representative. Paclitaxel is a widely used clinical anti-tumor drug, but its poor water solubility requires solubilizers such as Cremophor EL (polyoxyethylene castor oil) for dissolution. These solubilizers can easily cause severe adverse reactions such as allergic reactions [40]. To reduce toxic side effects, researchers have developed various nanoparticle formulations of paclitaxel. For example, Abraxane^®^ is an albumin-bound paclitaxel nanoparticle prepared by high-pressure homogenization technology, where paclitaxel binds to HSA through non-covalent interactions [41]. Abraxane^®^ was first approved by the FDA in 2005 for the treatment of breast cancer [42]. By leveraging the tumor-targeting properties of HSA, Abraxane^®^ can increase paclitaxel concentration in tumor tissues while reducing distribution in normal tissues, thereby decreasing toxic side effects. Clinical studies have shown that Abraxane^®^ demonstrates better efficacy in treating various tumors such as breast cancer and non-small cell lung cancer, with superior safety compared to traditional paclitaxel formulations [43]. In a Phase III clinical trial for breast cancer, compared to standard paclitaxel, albumin-bound paclitaxel significantly improved response rates (33% vs. 19%, respectively; *p* = 0.001) and prolonged time to tumor progression (23.0 vs. 16.9 weeks, respectively; hazard ratio = 0.75; *p* =0.006), indicating better efficacy compared to standard paclitaxel [44].

In addition to paclitaxel, doxorubicin, another broad-spectrum chemotherapeutic agent, has also achieved dual improvements in targeting and safety through the albumin prodrug strategy. Doxorubicin is an anthracycline anti-tumor antibiotic with broad-spectrum anti-tumor activity. However, its severe adverse reactions such as cardiotoxicity limit its clinical application [38]. To improve tumor targeting and reduce the toxic side effects of doxorubicin, researchers designed an HSA-based doxorubicin prodrug—Aldoxorubicin. Doxorubicin is conjugated to serum albumin through an acid-sensitive hydrazone linker. After enrichment in tumor tissues through pathways such as SPARC, it utilizes the weakly acidic tumor microenvironment to release active doxorubicin, enhancing local drug concentration and promoting the delivery of tumor-targeting drug payloads [45] while reducing toxicity to normal tissues such as the heart [46]. In a Phase II clinical trial, compared to doxorubicin, Aldoxorubicin significantly prolonged median progression-free survival in patients with advanced soft tissue sarcoma (5.6 [95% CI, 3.0–8.1] vs. 2.7 [95% CI, 1.6–4.3] months; *p* = 0.02), with no significant difference in median overall survival between the two groups. Independent evaluation showed that Aldoxorubicin had a higher overall tumor response rate than doxorubicin [47].

### 3.2. Targeted Therapy Prodrugs Based on HSA

Multiple receptors are abundantly expressed on tumor cell surfaces, critically regulating tumorigenesis, progression and metastasis and serving as pivotal targets for tumor-targeted therapy. Among these, folate receptor (FR) and CD44 represent two representative tumor-associated surface receptors with high expression and functional relevance in various malignancies.

Folate receptors (FRs) are frequently overexpressed on the surface of diverse tumor cells, especially in ovarian cancer, lung cancer and breast cancer [48]. As a classical target for active tumor targeting, FRs enable specific recognition and internalization by ligand-modified nanocarriers. Akbarian et al. prepared artemether (ARM)-loaded human serum albumin (HSA) nanoparticles via a desolvation method, improving the water solubility of ARM by 50-fold; they further fabricated folate-modified ARM-HSA nanoparticles, which exhibited enhanced targeting efficiency toward breast cancer cells with high FR expression [49].

Similarly, CD44, the primary receptor for hyaluronic acid (HA), is also overexpressed in a wide range of tumor cells and shares a tumor-targeting profile comparable to FRs. The specific binding between natural polymers and tumor-overexpressed membrane receptors constitutes the core mechanism of actively targeted nanoparticles. Taking advantage of the high affinity between HA and CD44, the assembly of hyaluronic acid with HSA-DOX nanoparticles generated a targeted delivery system with strong binding potential to CD44-overexpressing breast cancer cells [50].

Collectively, both folate receptors (FRs) and CD44 are highly expressed tumor surface markers, and their specific ligand–receptor interactions provide reliable strategies for the active targeting design of albumin-based nanoparticles.

Currently, a novel tumor-targeting molecule—aptamer (a single-stranded DNA or RNA molecule)—has emerged in clinical research due to its excellent specificity and affinity. Yang et al. [51] first investigated the role of protein conjugation strategies in improving the tumor targeting of aptamer AS1411. AS1411 is a 26-base guanine-rich aptamer with a unique G-quadruplex structure that can specifically target the nucleolin protein overexpressed on cancer cell membranes. During anticancer processes, it upregulates tumor suppressor gene p53 while downregulating oncogenes Bcl-2 and Akt1 by binding to nucleolin, thereby inhibiting cancer cell proliferation and inducing apoptosis. Pharmacokinetic analysis and tumor SPECT imaging studies show that the circulation half-life of HSA-AS1411 is more than 14 times that of AS1411, and the tumor uptake of HSA-AS1411 is superior to that of AS1411. The immunofluorescence staining of tumor tissues further demonstrates that due to prolonged circulation, AS1411 improves tumor retention. The results prove that conjugating HSA with aptamer AS1411 can significantly prolong AS1411’s circulation time and enhance aptamer tumor targeting.

### 3.3. Gene Therapy Prodrugs Based on HSA

Gene therapy utilizes genetic principles to introduce exogenous gene fragments such as DNA, antisense oligodeoxynucleotides (ODNs), messenger RNA (mRNA), and small interfering RNA (siRNA) to reverse the malignant phenotype of tumor cells and induce apoptosis.

However, the effectiveness of gene therapy in tumor suppression depends on the efficient delivery of gene drugs. RNA interference (RNAi) technology is a post-transcriptional tumor pathogenic gene silencing technique [52]. The specific mechanism involves the Dicer enzyme cleaving long-chain dsRNA into small interfering RNA (siRNA), or artificially synthesized siRNA, which enters the cytoplasm and binds to the RNA-induced silencing complex (RISC) to form an active complex. Subsequently, the Ago2 protein in RISC specifically recognizes and binds to target mRNA, and the nuclease activity of Ago2 in RISC cleaves the target mRNA, causing its degradation, thereby preventing mRNA from transcribing genes and translating into proteins, ultimately inhibiting target gene expression [53].

However, due to the unique structure of siRNA (negative charge, hydrophilicity, and high molecular weight), it cannot passively enter cells without protection. Therefore, constructing delivery carriers to protect siRNA and achieve targeted delivery to exert RNAi effects has become the primary goal [54]. Son et al. [55] found that thiolated HSA (THSA) and polymerized siRNA (psi) form stable nanosized complexes (psi-tHSAs) through chemical crosslinking and self-crosslinking. psi-tHSAs exhibit target gene silencing activity comparable to conventional complexes without significant cytotoxicity. The results demonstrate that HSA-siRNA nanomaterials have good therapeutic efficacy in tumor treatment. Ella et al. [56] synthesized siRNA–albumin complexes targeting MCL-1 (a driver of cancer treatment resistance). The results showed that siRNA conjugates bound to albumin were more effective in TNBC (triple-negative breast cancer) treatment than small molecule inhibitors of MCL-1, further demonstrating the feasibility of applying HSA-bound siRNA in tumor therapy. Sarett et al. [57] conjugated siRNA with diacyl lipid moieties (siRNA-L2), which can rapidly bind to albumin. Compared to unmodified siRNA, siRNA-L2 increased the circulation half-life by 5.7-fold and bioavailability by 8.6-fold and reduced renal accumulation. siRNA-L2 rapidly and uniformly penetrated tumor tissues, and 30 min after intravenous injection, siRNA-L2 achieved 99% tumor cell uptake.

### 3.4. Photothermal/Photodynamic Therapy (PTT/PDT) Prodrugs Based on HSA

Photodynamic therapy (PDT) is a precise treatment technology based on photochemical reactions. Its basic principle involves photosensitizers (PSs) being excited by specific wavelengths of light and interacting with molecular oxygen in target tissues to produce reactive oxygen species (ROS), leading to various biological effects such as cell death [58]. Photothermal therapy (PTT) is a non-invasive tumor treatment method that converts light energy into heat energy through photothermal agents (PTAs) under near-infrared light (NIR) irradiation, causing localized temperature increases to kill tumor cells [59]. The key to this treatment lies in the performance and targeting of photothermal agents to ensure heat is concentrated in tumor tissues while minimizing damage to normal tissues. Using PTT for tumor treatment offers minimally invasive properties, high selectivity, and combination therapy potential. Combining photothermal agents with HSA highlights targeting delivery advantages while also improving drug stability.

Temoporfin (mTHPC) is one of the most effective second-generation photosensitizers clinically used for photodynamic therapy (PDT) in head and neck squamous cell carcinoma [60]. mTHPC has promising applications, requiring less dosage, shorter treatment times, and lower light doses to achieve the desired PDT response. mTHPC exhibits an inherent lack of selectivity toward cancer cells. Its hydrophobic structure enables cellular entry mainly through passive diffusion and nonspecific lipoprotein binding, leading to widespread distribution in both tumor and normal tissues. Limited enrichment relies only on weak concentration differences, which fails to achieve precise tumor targeting and easily causes phototoxicity in normal tissues such as the skin. Human serum albumin (HSA) can improve the tumor selectivity of mTHPC via multiple mechanisms: first, achieving passive targeting and enrichment through the EPR effect in tumor tissues; second, mediating active endocytosis through gp60 and SPARC receptors highly expressed on tumor cells, thus preferentially entering cancer cells; third, ameliorating the hydrophobicity and aggregation of mTHPC to reduce nonspecific tissue deposition; and fourth, prolonging the systemic circulation half-life and enabling responsive drug release via the tumor microenvironment. Therefore, the HSA delivery system can significantly enhance the tumor-targeting selectivity of mTHPC and reduce damage to normal cells, providing an effective strategy for optimizing the targeted delivery of photosensitizers [61]. Researchers have developed a novel mTHPC formulation—mTHPC@HSA Complex—utilizing the multifunctionality of HSA as a “Trojan Horse” for drug delivery, enabling preferential accumulation in tumor tissues and reducing its phototoxicity [62].

IR780 is a lipophilic cationic near-infrared dye with tumor-targeting capabilities suitable for laser irradiation photothermal therapy. However, its lipophilicity and toxicity limit its application [63,64]. Studies have found that IR-780 can bind to plasma albumin and be released into the acidic microenvironment of ischemic tissues [65]. Based on this, researchers successfully prepared HSA-IR780 NPs for the combined photothermal and photodynamic therapy of cancer in vitro and in vivo. HSA-IR780 NPs improved the water solubility and optical stability of IR780 iodide in water and significantly reduced the toxicity of IR780 iodide in mice. Under certain conditions, HSA-IR780 NPs can simultaneously produce thermal effects and generate large amounts of ROS in vitro and in vivo, thereby severely damaging tumors [66].

### 3.5. Combination Therapy Strategies Based on HSA

**Figure 5:**
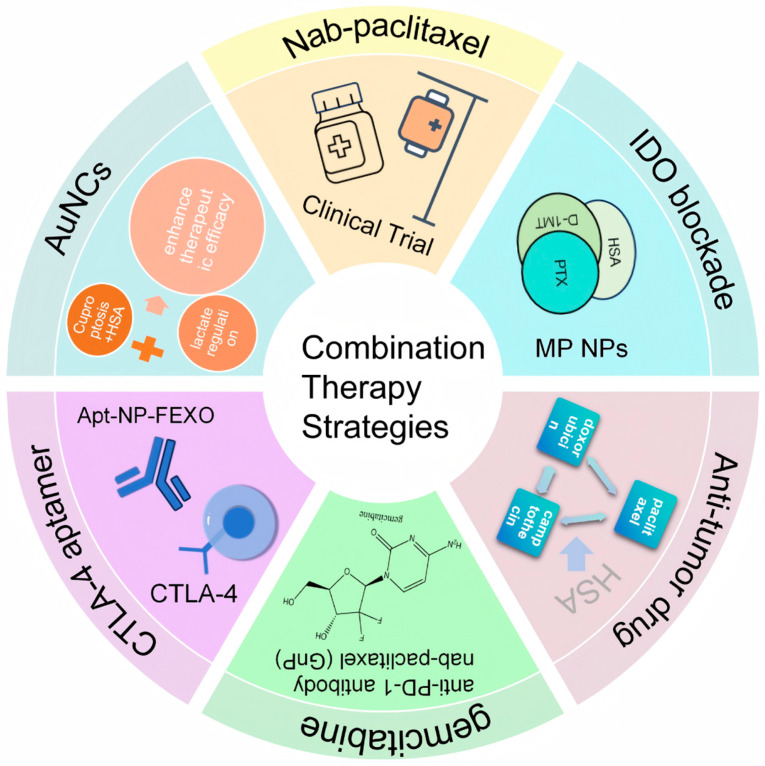
Combination therapy strategies. The diagram illustrates diverse combinatorial approaches to enhance antitumor efficacy, including clinically established regimens such as nab-paclitaxel-based therapies, and novel strategies combining HSA carriers with immunotherapies (e.g., PD-1/CTLA-4 checkpoint blockade), IDO inhibitors, chemotherapy (gemcitabine, doxorubicin), and advanced nanotherapies (AuNCs for cuproptosis-lactate regulation). These multi-modal strategies aim to overcome drug resistance, improve tumor targeting, and boost therapeutic outcomes through synergistic mechanisms.

Combination therapy strategies have gradually developed based on existing tumor treatment strategies, aiming to improve tumor treatment efficacy (Figure 5). Immune checkpoints have the function of regulating immune responses to prevent tissue damage and autoimmunity, which can be exploited by cancer cells to avoid destruction [67]. Therefore, immune checkpoint inhibitors (ICIs) targeting programmed cell death protein 1 (PD-1) have achieved a major breakthrough in the treatment of diverse solid tumors [68]. Attempts have already been made to combine PD-1 inhibitors with a nanoparticle albumin-bound paclitaxel for cancer therapy [69].

PD-1 inhibitor-based combination regimens have become a research focus, and the combination of PD-1 inhibitors with nanoparticle albumin-bound paclitaxel (nab-paclitaxel) has been explored for cancer therapy [69]. As a novel taxane agent, nab-paclitaxel exhibits favorable solubility, bioavailability and safety profiles relative to conventional taxanes, laying a foundation for its combination with immunotherapy [69].

For soft tissue sarcoma (STS), complete resection is the first-line treatment for early-to-intermediate disease, while ~50% of patients develop lung metastasis [70,71]. Advanced STS is managed with doxorubicin (first-line) and docetaxel plus gemcitabine (second-line), yet median overall survival remains only ~12 months, highlighting an urgent need for more effective strategies.

The combination of nab-paclitaxel and PD-1 inhibitors has demonstrated promising anti-tumor activity across multiple malignancies. This regimen is well-tolerated overall, with no dose reductions in either agent due to adverse events or treatment-related deaths reported [69]. As a potential therapeutic option for advanced STS, this PD-1-based combination still requires randomized controlled trials to validate its efficacy and optimal administration protocols. Given the clinical maturity of nab-paclitaxel, its combinations with other anticancer agents are also under active clinical investigation.

Zafar et al. [72] found that cuproptosis is closely related to immunogenic cell death (ICD), which stimulates anti-tumor immune responses and promotes dendritic cell (DC) maturation and CD8+ and CD4+ T cell maturation. Moreover, lactate regulation is also closely related to tumor immunity. Elevated lactate levels hinder anti-tumor immune responses. By combining cuproptosis and lactate regulation to enhance therapeutic efficacy, researchers designed a cuproptosis–lactate regulation therapeutic nanosystem (BAu-CuNCs). First, Cu was bound to HSA, then copper–HSA was further bound to AuNCs, and bacterial membrane coating was applied to the outer surface to produce BAu-CuNCs. The cuproptosis effect induces ICD, thereby activating CD8+ tumor immunity and CD4+ immune T cells. Simultaneously, AuNCs promote excessive ROS production in tumor cells under near-infrared conditions, blocking glycolytic metabolic pathways and ultimately reducing lactate and ATP levels. The simultaneous reduction in lactate and ATP levels and GSH depletion significantly enhances tumor cell responsiveness to cuproptosis, thereby enhancing the therapeutic potential of this treatment approach.

Pancreatic cancer is considered one of the most aggressive and lethal forms of cancer due to its tendency to rapidly spread to other parts of the body, lack of obvious symptoms in early stages, and resistance to traditional chemotherapy. Chen Zhitao et al. [73] retrospectively analyzed clinical data from 27 patients with advanced pancreatic cancer treated with an anti-PD-1 antibody combined with gemcitabine plus nab-paclitaxel (GnP) regimen, evaluating treatment safety as well as the objective response rate (ORR), disease control rate (DCR), progression-free survival (PFS), and overall survival (OS). Regarding safety, most adverse events were mild to moderate, with a low incidence of severe adverse events (SAEs) and good overall tolerability. Combination therapy showed significant anti-tumor activity, especially in patients with high PD-L1 expression or high tumor mutational burden (TMB). However, this study was a retrospective clinical study with a small scale and sample size, requiring further research to determine the therapeutic effects of PD-1 inhibitors combined with albumin in pancreatic cancer.

Triple-negative breast cancer (TNBC), a solid tumor with high distant recurrence rates and low overall survival rates, accounts for 15–20% of all breast cancers. Studies show that combination therapy using paclitaxel, camptothecin, and doxorubicin to induce immunogenic cell death (ICD) appears to be a good treatment option, but problems with high toxic side effects remain. Matilde Tubertini’s team found through in vitro experiments that by making HSA into nanoadhesives for combined cancer immunotherapy, ICD can be achieved, and IDO1 enzyme immunosuppression can be alleviated, thereby reducing toxicity to the body [74].

Indoleamine-2,3-dioxygenase (IDO), an immunosuppressive enzyme, has become a potential target for cancer immunotherapy. However, monotherapy with IDO inhibitors has significant limitations in clinical practice. The authors of [75] synthesized a drug conjugate, melatonin–paclitaxel (MP), by linking the IDO inhibitor D-1-methyltryptophan (D-1MT) with the chemotherapeutic agent paclitaxel (PTX) via an ester bond. MP exhibits a microtubule-stabilizing effect similar to that of PTX. MP can bind to human serum albumin to form albumin-bound MP nanoparticles (MP NPs) with a particle size of ~115 nm. MP NPs significantly increase the tumor concentration of D-1MT by hydrolyzing MP in the tumor. These results verify the anti-tumor effect of the dual-drug combination system constructed from IDO inhibitors and PTX, showing promise for clinical translation.

The CTLA-4 (Cytotoxic T-Lymphocyte-Associated Protein 4) aptamer was conjugated to the surface of albumin nanoparticles, forming an aptamer-modified nanostructure (Apt-NP). To enhance the efficacy of immune checkpoint blockade therapy, an antihistamine, fexofenadine, was further combined and encapsulated into Apt-NP, resulting in drug-loaded nanoparticles (Apt-NP-FEXO). The anti-tumor immune effect of Apt-NP was superior to that of the free CTLA-4 aptamer, while Apt-NP-FEXO demonstrated even greater anti-tumor efficacy in vivo compared to Apt-NP [76].

## 4. Challenges and Limitations

After conjugation with drugs, drug release from human serum albumin is influenced by multiple factors. The release efficiency of some albumin prodrugs at tumor sites is affected by nanoparticle surface modifications, the tumor microenvironment, and the stability of drug–albumin conjugation. If the conjugation is unstable, it leads to low release rates at tumor sites, affecting treatment efficacy. More importantly, premature release before reaching the target site can cause toxic side effects in other normal tissues and organs. Research has improved drug release rates by designing pH-sensitive or reducible enzyme-sensitive linkers. In short, drug–carrier complexes release responsively under specific microenvironmental conditions. However, nanocarriers responding to single stimuli (such as pH or enzymes) cannot overcome multiple physiological barriers and are prone to off-target effects due to similar stimuli in normal tissues. With continuous advances in biomedical nanotechnology, stimulus-responsive DDSs (drug delivery systems) have partially solved nanoparticle delivery obstacles.

Although smart responsive delivery systems alleviate the above release-related drawbacks, the intrinsic biological complexity of tumors still poses severe challenges to precise albumin-based drug delivery. Due to tumor heterogeneity, tumor cells exhibit diversity in genes, phenotypes, functions, and microenvironments, directly leading to a series of chain reactions such as drug resistance and recurrence in tumor treatment. Even though tumor targeting attracts nanocarriers to become enriched in tumor tissues, different organisms express different HSA-binding proteins in tumors, indirectly causing differences in the accumulation capacity of the same drug delivery carrier system at specific target sites in different environments, affecting treatment efficacy.

Besides molecular and cellular heterogeneity within tumors, the unique physical microstructure of solid tumors also severely weakens the delivery efficiency of albumin nanoparticles. Dense tumor stroma exists in most human solid tumors (such as bone, bladder, brain, and ovary), causing the EPR effect to fail. The co-delivery of synergistic drugs by nanoparticles requires the simultaneous delivery of different drugs to heterogeneous cell populations (such as tumor stem cells and immune cells), but traditional drug delivery systems cannot achieve spatiotemporally controlled differential release.

## 5. Future Perspectives

In recent years, research on albumin-based anti-tumor prodrugs has made significant progress, primarily focusing on combination therapy, targeted therapy, responsive release, and drug delivery. As a natural drug delivery carrier, human serum albumin plays an important role in improving drug properties. Dou et al. studied albumin-binding NQO1 (quinone oxidoreductase 1) bioactivatable drugs, which can significantly inhibit tumor growth in pancreatic cancer mouse models, representing a potential innovative therapy for pancreatic cancer [77]. The chemical modification of human serum albumin to improve its targeting is also an important direction in modern biopharmaceutics. Koichi et al. fused CBD (collagen-binding domain of von Willebrand factor) with HSA to obtain CBD-SA, which retains passive targeting and can also bind to collagen in tumor stroma after tumor vascular extravasation. Through the combined action of these two modes, the ability of HSA to be used in tumor-targeted therapy and drug therapeutic effects are enhanced [37]. In contrast, Dox-AlbCages rely on albumin biomineralization and size-controlled nanocage structures to achieve superior intracellular drug uptake and responsive controlled release, which differs greatly from the stroma-targeting mechanism of CBD-SA. Regulating albumin nanosize to optimize blood circulation and tumor penetration provides personalized approaches for size-dependent tumor therapy. Researchers have proposed doxorubicin albumin nanocages (Dox-AlbCages) for treating aggressive cancers. Through albumin-templated biomineralization, doxorubicin is precipitated within albumin nanocages to precisely prepare doxorubicin–albumin cages with controllable particle sizes. Dox-AlbCages show significantly increased cellular uptake and responsive intracellular release, achieving controlled drug release and more effective tumor cell inhibition [78]. Meanwhile, researchers have turned their attention to modular drug design, which has an extended circulation half-life and programmable multifunctionality [79].

To summarize, NQO1 bioactivated albumin prodrugs achieve precise tumor treatment relying on enzyme-specific activation in tumor microenvironments; CBD-SA realizes dual passive and tumor stromal active targeting through structural fusion modification; and Dox-AlbCages depend on an albumin biomineralized nanocage structure to realize efficient intracellular drug delivery and stimuli-responsive release. These three albumin-based tumor therapeutic systems adopt completely different mechanisms including enzyme activation, stromal targeted binding and nanoscale structural regulation, which jointly enrich the diversified development strategies of HSA anti-tumor prodrugs.

## 6. Conclusions

Human serum albumin (HSA) has emerged as a promising carrier for prodrug delivery in tumor therapy due to its unique biological properties, including excellent biocompatibility, prolonged circulation half-life mediated by the FcRn recycling mechanism, and inherent tumor-targeting capabilities via the passive EPR effect and active targeting through Gp60 and SPARC receptors. The clinical success of Abraxane and the development of other HSA-based prodrugs for chemotherapy, targeted therapy, gene therapy, and phototherapy demonstrate the versatility and potential of HSA as a drug delivery platform. Despite challenges such as drug release regulation, tumor heterogeneity, and dense tumor stroma, ongoing research into smart responsive systems, combination therapies, and novel HSA modifications offers promising solutions. Future directions include the development of more sophisticated targeting ligands, stimuli-responsive linkers, and personalized nanomedicine approaches based on HSA. As our understanding of HSA’s role in drug delivery continues to evolve, it is anticipated that HSA-based prodrugs will play an increasingly important role in the clinical management of cancer, offering improved therapeutic outcomes with reduced side effects.

## Figures and Tables

**Table 1:** HSA binding sites and characteristics.

Binding Site	Binding Domains	Binding Features
Sudlow Site I	Subdomain IIA	Combined with large-volume and rigid aromatic drugs (such as warfarin, azacitidine), which have strong hydrophobicity and deep cavity structures.
Sudlow Site II	Subdomain IIIA	Combined with small molecule carboxylic acid drugs (such as ibuprofen, diazepam), where hydrophobicity and polarity interact simultaneously, and the binding site is relatively shallow.
Cys-34	Subdomain IA helix2-helix3	Highly selective binding to metal ions, with a free thiol group (-SH), which remains in a reduced state under physiological conditions and has antioxidant properties.
FA1	Subdomain IB	Low-affinity fatty acid binding site, mainly binds medium/long-chain fatty acids at high concentrations.
FA2	Subdomain IA-IIA	High-affinity fatty acid binding site, contributes to the initial binding of long-chain fatty acids.
FA3	Subdomain IIIA	Medium-affinity site, overlaps with Sudlow Site II, competitive binding with drugs.
FA4	Subdomain IIIA	High-affinity site, overlaps with Sudlow Site II, key site for long-chain fatty acid binding.
FA5	Subdomain IIIB	Highest-affinity site, the primary binding site for long-chain fatty acids.
FA6	Subdomain IIA-IIB	Medium/low-affinity site, functions as a “buffer pool” to prevent free fatty acid toxicity.
FA7	Subdomain IIA	Low-affinity site, binds excess fatty acids, regulates the total fatty acid binding capacity of HSA.

## Data Availability

Data sharing is not applicable to this article as no new data were created or analyzed in this study.
